# The effects of transcranial direct-current stimulation (tDCS) on pain intensity of patients with fibromyalgia: a systematic review and meta-analysis

**DOI:** 10.1186/s12883-023-03445-7

**Published:** 2023-11-02

**Authors:** Reza Moshfeghinia, Dorsa Shekouh, Sara Mostafavi, Mehrnaz Hosseinzadeh, Amir Reza Bahadori, Saeed Abdollahifard, Ali Razmkon

**Affiliations:** 1 Student Research Committee, Shiraz University of Medical Sciences, Shiraz, Iran; 2 Research Center for Neuromodulation and Pain, 4th floor, Boghrat building, Zand Street, Shiraz, Iran; 3 Fasa Neuroscience Circle (FNC), Student Research Committee, Fasa University of Medical Sciences, Fasa, Iran; 4 School of Medicine, Shiraz University of Medical Sciences, Shiraz, Iran

**Keywords:** Fibromyalgia, tDCS, Pain, Transcranial Direct Current Stimulation, Noninvasive brain stimulation

## Abstract

**Introduction:**

Fibromyalgia (FM) is a chronic pain condition that affects millions of people worldwide. Transcranial Direct Current Stimulation (tDCS) is a non-invasive brain stimulation technique that has shown promise as a potential treatment for FM by modulating pain perception and reducing symptoms, such as fatigue and depression. We aimed to systematically review studies that assess the effect of tDCS on pain reduction in FM patients.

**Methods:**

Seven electronic databases (PubMed, Scopus, Embase, PsycINFO, Web of Science, Cochrane, and CINAHL Complete) were searched for records in English. Studies that measured the effect of tDCS on pain intensity in FM patients were included. The Cochrane Collaboration’s tool was used to assess the quality of the included studies. A random-effect model was preferred, and statistical analysis was performed by Stata software version 17.

**Results:**

Twenty studies were included for qualitative, and eleven for quantitative analysis. Out of 664 patients included in the study, 443 were in the stimulation group. The left M1 area was the most common stimulation target (*n* = 12), and 2 mA was the most common stimulation amplitude (*n* = 19). The analysis showed that active tDCS significantly reduced pain intensity in FM patients in comparison to the sham group (SMD= -1.55; 95% CI -2.10, -0.99); also, no publication bias was noted.

**Conclusion:**

Our systematic review highlights the potential effect of tDCS on the reduction of pain intensity in FM patients. Additionally, this current evidence could suggest that tDCS applied at an intensity of 2mA to the left M1 is the most effective strategy.

**Supplementary Information:**

The online version contains supplementary material available at 10.1186/s12883-023-03445-7.

## Introduction

 Fibromyalgia (FM) is a heterogeneous, long-lasting disorder that mostly presents with widespread musculoskeletal pain [1]. The prevalence of FM varies across different regions and populations, but the global average is estimated to be around 2.7% [2]. Due to the debilitating pain and concomitant symptoms such as fatigue and cognitive impairments, FM impairs psychological, physical, and social functioning; therefore, it may result in mental health issues for affected individuals [3, 4]. Various interventions, such as exercises, cognitive-behavior therapy (CBT), medications, and neuromodulation, have been proposed for the treatment of FM. Both pharmacological and non-pharmacological treatments can help relieve pain in FM patients [5]. Pharmaceutical treatment is used widely owing to availability and accessibility; however, it only relieves the patients’ condition and does not cure FM [6–8]. Some of the common medications that are prescribed to reduce the symptoms of patients are gamma-aminobutyric acid A agonists, Benzodiazepines, selective serotonin reuptake inhibitors (SSRI), and serotonin-norepinephrine reuptake inhibitors (SNRI). In addition to therapeutic effects, the aforementioned pharmaceutical drugs may have many side effects such as erectile dysfunction, dizziness, gastrointestinal discomfort, and tiredness [6].

Although there is no clear understanding of the disease’s etiology, the consensus on its pathogenesis is dysfunctions in the central processing of pain perception and control systems that result in a state of increased sensitization to pain and other stimuli [9]. One possible way of ameliorating the FM symptoms may be to modulate the activity of brain areas involved in pain perception and control mechanisms through non-invasive brain stimulation techniques. Neuromodulation, especially transcranial direct current stimulation (tDCS), has been shown to have a remarkable impact on pain relief and functional improvement of FM patients in many studies [6, 10, 11]. Furthermore, the efficacy of tDCS in various psychological conditions such as depression, tinnitus, and pain reduction has been demonstrated and might be effective for reducing the symptoms of patients with FM [6, 8, 10]. However, the results have been inconsistent and heterogeneous, making it difficult to draw definitive conclusions about the efficacy and optimal protocol of tDCS for FM. A systematic review and meta-analysis by Hou et al. reviewed 5 articles on the effect of tDCS on FM and found a significant result supporting its analgesic effects but with a smaller effect size than rTMS. They also found no substantial difference in effect size between M1 and DLPFC as target sites [12]. However, Zhu et al. found M1 as the effective target area but did not confirm the role of DLPFC stimulation in pain reduction of patients with FM [13]. A recent meta-analysis by Teixeira et al. included 16 RCTs that encompassed 26 different tDCS protocols and confirmed the overall analgesic effect of the intervention and the effectiveness of targeting both M1 and DLPFC [14].

Previous meta-analyses and systematic reviews indicated that tDCS is useful for reducing pain intensity in FM patients. However, previous studies showed that the impact of tDCS depends on the location of its anodal placement, amplitude, the duration of each session, frequency, and other variables. Therefore, we decided to update previous meta-analyses to gain a better understanding of the effect of tDCS on the pain intensity of FM patients and the optimal protocol.

## Methods

To establish the effects of transcranial direct current stimulation on pain intensity perception in patients with FM, this systematic review and meta-analysis were conducted according to the Preferred Reporting Items for Systematic Reviews and Meta-analysis (PRISMA) guidelines for 2020 [15]. The study protocol was registered in the International prospective register of systematic reviews (PROSPERO) with the following registration number: CRD42022383060.

### Search strategy

Seven electronic databases (PubMed, Scopus, Embase, PsycINFO, Web of Science, CINAHL Complete, and Cochrane) were searched for English records up to June 2022. Searches were performed using combinations of the following keywords: “Fibromyalgia” OR “Fibrositis” AND “Transcranial Direct-Current Stimulation” OR “tDCS”. The search didn’t limit the above words or any synonyms included in the search strategy. Detailed search strategies for each database are accessible in Supplementary Materials Part B. The references of included studies were also screened to identify potentially eligible articles.

### Eligibility criteria

We included clinical trial studies that investigated the effect of tDCS on the pain intensity of FM in humans older than 18 years with symptoms lasting more than 3 months. The following studies were excluded: (1) studies that examined pain in conditions other than FM; (2) studies with insufficient data to calculate the effect of tDCS on pain intensity in FM patients; (3) duplicate studies or studies with overlapping participants; (4) observational studies, reviews, editorials, conference papers, case series/reports with fewer than 10 cases, and animal experiments; (6) qualitative designs. Also, for the meta-analysis, studies without control or placebo groups were excluded. Studies were identified by two investigators (DSh and SM) independently according to the above criteria, while discrepancies were resolved by consensus or with a third investigator (RM or SA).

### Study selection

Two authors (DSh and SM) independently screened the titles and the abstracts of the potentially eligible studies using EndNote software version 20. They applied the predefined inclusion and exclusion criteria to select the studies for full-text assessment. The full texts of the selected studies were retrieved and evaluated independently by the same authors. Any conflicts related to the study design or methods, and the final decision of including or excluding studies, were resolved by two other authors (RM and SA). At all these stages, a functional neurosurgeon (AR) was consulted if necessary. The number of studies that were included and excluded at each stage was recorded and reported in a PRISMA flow diagram.

### Data extraction

Two authors (DSh and SM) separately extracted the information from included articles. Disagreements were resolved by discussing the controversies with a third author (SA). The following general characteristics were collected from each study: First author, publication year, country, study type, sample size, target areas and electrode positions, tDCS protocol (intensity, session duration, number of sessions, and duration of intervention (wks)), control condition, associated interventions, and pain intensity outcome measurement.

### Risk of bias assessment

We assessed the risk of bias of included studies with the risk of bias assessment tool of the Cochrane Collaboration [16]. Two reviewers (DSh and SM) independently assessed the risk of bias in the studies. Studies were judged individually as three grades: ‘low risk’ was assigned if the study addressed risks well because the study design was clarified, ‘unknown risk’ was assigned if it retained risks because details were not stated suitably, and ‘high risk’ was allocated if there were serious risks that could affect the study outcome due to biased study design. If an agreement could not be reached, a third reviewer (RM or SA) acted as an arbiter.

### Quantitative analysis

The mean changes and standard deviation (SD) of pain intensity in the tDCS and Sham groups were used to obtain the overall effect size (standardized mean difference (SMD)). We also calculated SD using the standard error (SE) and 95% confidence interval (CI) through a method described by Hozo et al. [17]. A random-effects model was used to pool the extracted unstandardized difference in means and the corresponding confidence intervals of the studies. Heterogeneity among the studies was assessed using the chi-squared test and I2 statistic. To assess the risk of publication bias, we employed two statistical tests: Egger’s test and Begg’s test. These tests examine the relationship between the effect size and the standard error or the sample size of each study and provide a *p*-value to indicate the significance of the asymmetry. To visualize the publication bias, a funnel plot was utilized, plotting the effect size against the standard error for each study. A symmetrical funnel-shaped distribution of the studies suggests a low risk of publication bias, while an asymmetrical distribution suggests a high risk of publication bias. Subgroup analysis was performed to estimate the pooled effect in the target population, type of the study, pain assessment tools, current intensity, electrode site, and sex subgroups. A sensitivity analysis was also conducted to test the robustness of the pooled effect size. All analyses were conducted in Stata software (version 17, Stata Corporation, College Station, Texas, USA). *P*-values less than 0.05 were considered statistically significant.

## Results

### Selection of studies

**Fig. 1:**
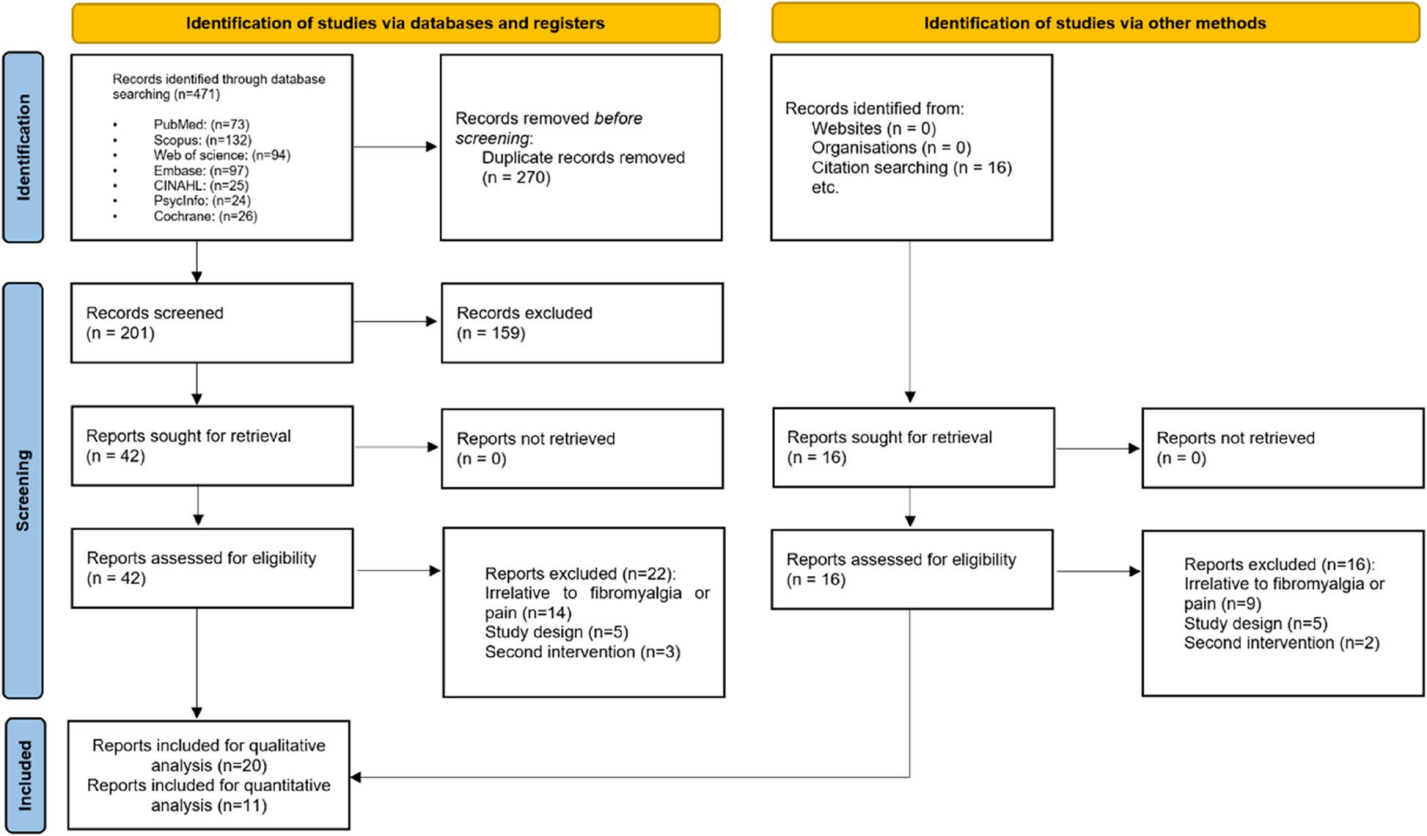
PRISMA flowchart of the included studies

Figure 1 depicts the PRISMA flow diagram. The search criteria initially yielded 471 articles from the databases based on the proposed keywords. EndNote automatically removed 270 duplicates, and 159 articles were subsequently excluded after screening the titles and abstracts. Consequently, 42 articles were included in this screening step. Following full-text evaluation, 22 articles were excluded, ultimately leaving 20 studies for qualitative analysis and 11 studies for quantitative analysis.

### Study characteristics

We included 16 RCTs and four crossover studies, with a total of 664 participants. Of those, 443 were in the active stimulation group. The studies used anodal tDCS with different intensities, electrode positions, and stimulation durations: Anodal tDCS was administered at an intensity of 2 mA for 17 studies [6, 8, 18–32], 1.5 mA for 2 studies [6, 33], and 1 mA for 2 studies [34, 35]. The locations of the target electrode were the left primary motor cortex (M1, corresponding to C3) with an anode over the left M1 [8, 18, 19, 21, 22, 25, 26, 28–30, 32, 34], an anode over left C2 [33], anode over right C2 [24], an anode over left DLPFC [21, 23, 27, 28, 31–33, 35], an anode over the right occipital nerves [6], an anode over SO [19, 20], an cathode over SO [19], cathode over M1 [19]. In all studies except one [31], stimulation was applied for 20 min, although the number of sessions mostly varied from 1 to 10, except for 2 studies, one of which used 20 sessions [23], and the other was a home-based study that applied the stimulation for 20–60 sessions [31], with a mean of 6.68 sessions (excluding the study with 20–60 sessions). For assessing pain intensity during the intervention, the studies used different scales. Four studies used the Numeric Rating Scale (NRS) [6, 18, 24, 33], 2 studies assessed pain using the Visual Analogue Scale (VAS) [8, 19–22, 25–32, 34], and the Pain Catastrophizing Scale (PCS) was used in two of them [23, 35] (Table 1).

**Table 1:** Characteristics of all included studies

Author, publication year	country	design	Active group (n)	Sham group (n)	active electrode location	Cathode location	tDCS protocol	Associated interventions/ form of tDCS	Side effects	Pain outcome used	Notes (aim, result, duration of effects last)
qualitative											
Roizenblatt et al., 2007 [32]	Brazil	RCT	11 (f)		M1	Right SO	Anodal, 2 mA, 20 min, 5 sessions in consecutive days	None	None	VAS	Aim: to investigate the effect of tDCS-induced pain reduction on sleep structure in FM Result: tDCS stimulation can decrease pain only in M1 condition, and tDCS can change sleep structure, specific to the site of stimulation. Duration of effect: NA
			11 (f)		Left DLPFC						
				10 (f)	sham		Turned off after 30 s of stimulation				
Silva et al., 2017 [35]	Brazil	RCT, cross-over design	17/20 (f)	18/20 (f)	Left DLPFC	Right SO	Anodal, 1 mA, 20 min, Single session	Go/No-go task	Minor: tingling, burning, and itching. In both conditions	HPTh, HPTo,	Aim: Anodal tDCS over the left DLPFC modulates attention and pain in fibromyalgia Result: active stimulation increased HPTh and HPTo Duration: NA
Mendonca et al., 2016 [20]	Brazil	RCT	30 - f/m (tDCS/AE group (*n* = 15), tDCS group (*n* = 15))	15 (AE group)	Left M1	Right SO	Anodal, 2 mA, 20 min, 5 sessions, consecutive days over the first week.	AE on a treadmill, 40 min, 9 sessions over 4 weeks	Mild adverse effects, not different between groups.	VNS	Aim: to assess the effect of combined intervention of tDCS and AE on pain in FM Result: the combination intervention is superior in pain reduction compared to individual treatments. Duration of effect: after one month of intervention.
De Ridder et al., 2017 [24]	Belgium	RCT, crossover design	19	19 matched healthy control (Crossover design, 2 weeks washout period between conditions)	Right Occipital nerve field (OCF)	Left OCF (C2 dermatome)	Anodal, 1.5 mA, 20 min, 3 sessions, every two days, over a week	None	None	NRS, PCS	Aim: to investigate the mechanisms behind the effect of OCF on pain in FM. Result: active tDCS shows significant pain reduction compared to sham and baseline. Duration of effects: NA
							Sham				
Brietzke et al., 2020 [31]	Brazil	RCT	10 (f)	10 (f)	Left DLPFC	Right DLPFC	Anodal, 2 mA, 30 min, 60 sessions over 12 weeks (5 consecutive days minimum interval of 16 h)	None/ bifrontal HB-tDCS	Mild and transient: headache, itching, tingling, and local redness	VAS (global pain in last 24 h), B-PCP:S, PPT, HPTo,	Aim: To test the effectiveness of HB-tDCS over DLPFC in multiple sessions on daily pain scores. Result: tDCS was superior to the sham group alone in reducing pain intensity. Levels of BDNF predicted better response Duration of effect: NA
Kang et al., 2020 [29]	South Korea	RCT	46	No sham group	Left M1	Right SO	Anodal, 2 mA, 20 min, 5 sessions, consecutive days.	pharmacotherapy	No serious adverse effect was reported.	VAS	Aim: to investigate the effects of add-on tDCS stimulation on pain in FM. Result: tDCS is effective in pain reduction and other features in FM. Duration of effects: after one month post-stimulation.
Forogh et al., 2021 [27]	Iran	RCT	15 (f)	No sham group	Left DLPFC	Right SO	Anodal, 2 mA, 20 min, 3 sessions, over one week (every other day)	None	None	VAS	Aim: to compare the effects of rTMS and tDCS on pain and quality of life in FM Result: pain intensity was significantly reduced in both groups, however, rTMS was more effective. Duration of effect: just after course termination
EL-Badawy et al., 2021 [25]	Egypt	RCT	15	No sham group – but the TMS group with *n* = 15 was examined	Left M1	Right SO	Anodal, 2 mA, 20 min, 8 sessions,	None	Local tingling, well-tolerated complaint of headache and dizziness	VAS	Aim: to compare the efficacy of rTMS and tDCS in pain reduction in FM Result: both interventions were significantly efficient in pain reduction significantly, however, rTMS resulted in better improvement. Duration of effects:
Caumo et al., 2022 [23]	Brazil	RCT	24 (f)	24 (f)	Left DLPFC	Right DLPFC	Anodal, 2 mA, 20 min	None/ bifrontal HB- tDCS	Severe: burning sensation, Mild: tingling, redness, headache, neck pain, mood swings, concentration difficulties	VAS, PCS, FIQ	Aim: to evaluate the efficacy and safety of home-based bifrontal tDCS in reducing pain and disability due to pain in FM Result: positive effect Duration of effects: NA
quantitative											
Fregni et al., 2006 [28]	Brazil	RCT						None	Mild, similar to sham: Sleepiness and headache were the most frequent	VAS	Same as study subjects in the Roizenblatt et al. study Result: tDCS stimulation of M1 had significant pain reduction in FM compared to DLPF and sham group Duration of effect: at least 3 weeks after stimulation
Valle et al., 2009 [21]	Brazil	RCT	14 (f)		M1	Contralateral SO	2 mA, 20 min, 10 sessions, consecutive days	None	Minor and uncommon: tingling, skin redness. Same with the sham group	VAS	Aim: to investigate the effect of tDCS stimulation of M1 and DLPFC on FM Result: M1 stimulation markedly decreases pain in FM but no evidence for the efficacy of DLPFC stimulation Duration of effect: M1 stimulation reduces pain that persists for up to 2 months.
			13 (f)		DLPFC						
				14 (f)	Sham (M1)						
Mendonca et al., 2011 [19]	Brazil,	RCT	6		Right SO	Left M1	Cathodal, 2 mA, 20 min	None	Mild tingling at the beginning, no side effects	VNS, PPT, total body area of pain	Aim: to determine the efficacy of tDCS with different active electrode positions on pain reduction in FM Result: SO tDCS resulted in a significant pain reduction both as cathode and anode Duration of effects: NA
			6		Left M1	Right SO					
			6		Left M1	Right SO	Anodal, 2 mA, 20 min				
			6		Right SO	Left M1					
				6	Left M1	Right SO	Sham, current on only for the initial 30 s				
Villamar et al., 2013 [22]	USA	RCT, crossover design	16/18	Crossover design, participants have 3 different interventions with 7 days interval	Left M1		Anodal, 2 mA, 20 min, 1 session	None /HD-tDCS	Mild to moderate tingling or itching during both active and sham stimulation, which resolved over a few minutes	VNS, PPT, and others	Aim: short term effects of HD-tDCS on pain reduction in FM Results: Immediately after stimulation only cathodal HD-tDCS was effective and 30 min after stimulation both active interventions resulted in better pain reduction than the sham. Duration of effects: NA
							Cathodal, 2 mA, 20 min, 3 sessions				
							sham				
Foerster et al., 2015 [26]	USA	RCT, crossover design	12 (f)	Crossover design, 7 days washout period between conditions	Left M1	Right SO	Active Anodal, 2mA, 20 min, 5 consecutive days	None	None	VAS	Aim: To investigate the effects of tDCS on brain metabolites and the predictive value of treatment efficacy in FM. Result: tDCS reduced pain intensity. tDCS also have effects on the brain metabolites. Baseline levels of these metabolites predicted pain reduction after tDCS. Duration of effects: NA
							sham				
Fagerlund et al., 2015 [18]	Norway	RCT	24	24	Left M1	Right SO	Anodal, 2 mA, 20 min, 5 sessions, consecutive days	None	Skin redness, sleepiness, and tingling were reported same in the active and sham group. Acute mood changes were more reported in the sham group	VAS, PPT	Aim: to investigate the effect of tDCS stimulation on pain in FM Result: tDCS has a small but significant effect on pain reduction in FM.Duration of effects: NA
Junior et al., 2015 [34]	Brazil	RCT	10 (f)	10 (f)	Left M1	Right SO	Anodal, 1 mA, 20 min, 10 sessions, consecutive days.	None	None	VAS	Aim: to evaluate the effect of tDCS on pain and quality of life in FM. Result: tDCS is effective in pain control of FM. patients Duration of effects: NA
Yoo et al., 2018 [6]	Belgium	RCT	20		Left DLPFC + ONS	Right SO	1.5 mA, 20 min, each intervention, 8 sessions over 4 weeks, sessions were 3 days apart.	ONS	Tingling and itching	NRS	Aim: to investigate the effect of adding prefrontal tDCS before ONS on pain and quality of life in FM. Results: prefrontal tDCS did not change the pain compared to ONS-only group. Duration of effects: NA
			20		ONS alone						
				20							
To et al., 2017 [33]	Belgium	RCT	11		Left DLPFC	Right DLPFC	Anodal, 1.5 mA, 20 min, 8 sessions, in 4 weeks	None	None	NRS, PCS	Aim: to compare the effects of bifrontal and occipital tDCS on pain and fatigue in FM Result: both bifrontal and occipital tDCS reduced pain scores, DLPFC also improved fatigue and provided more general relief than C2 stimulation Duration of effects: NA
			15		Left occipital	Right occipital					
				16			Initial 10 s of active stimulation and then inactive for 20 min, same number of sessions.				
Khedr et al., 2017 [30]	Egypt	RCT	18	18	Left M1	Right SO	Anodal, 2 mA, 20 min, 10 sessions, 5 consecutive days over two weeks.	None	Itching and redness of skin in only 3 cases from the active group.	VAS	Aim: to evaluate the effects of tDCS on pain, mood, and serum endorphin levels in the treatment of FM. Results: M1-tDCS was able to improve pain in FM significantly. This effect is related to changes in serum endorphin levels Duration of effects: at least one month after stimulation
Melo et al., 2020 [8]	Brazil	RCT	11 (f)		Left M1	Right SO	Anodal, 2 mA, 20 min, 1 week (5 consecutive days)	None	None	VAS	Aim of study: To compare the effects of two tDCS protocols on pain and EEG alpha-2 oscillations in FM Result: Both protocols reduced pain intensity without significant difference, but only those received for 5 consecutive days, showed a significant reduction in alpha-2 power in the frontal and parietal region Duration of effects: NA
			9 (f)				Anodal, 2 mA, 20 min, 2 weeks (10 consecutive days excluding weekends)				
				11 (f) − 5 consecutive days							

*Abbreviations*: *HPTh *Heat pain threshold, *HPTo *Heat pain tolerance, DLPFC, *SO *Supraorbital, *VAS *Visual analog scale, *FIQ *Fibromyalgia impact questionnaire, *B-PCP:S *Brazilian Portuguese version of the profile of chronic pain: screen, *PPT *Pain pressure threshold, *HB-tDCS *home-based tDCS, *CIRS *Cumulative illness rating scale, *VNS *Visual numerical scale, *DASS-21 *Depression anxiety stress scale-21, *AE *Aerobic exercise, *ONS *Occipital nerve stimulation, *PCS *Pain catastrophizing scale

### Side effects

We assessed the reported adverse effects of tDCS, and most of the studies reported no significant or only mild adverse effects; tingling or itching were the most common. Six studies [19, 24, 26, 29, 31, 32] reported the severity of side effects as major; the mentioned major side effects were skin redness [18, 23, 30], sleepiness [18, 28], tingling [18, 23, 25], burning [23], headache [28], and itching [32]. Five studies reported mild side effects, such as skin redness [20, 21], tingling [21, 22, 35], itching [22, 35], dizziness [29], light headache [29], transient sleep disturbances [29], and burning [35].

### Second intervention

Four of the included studies have investigated the effects of adding a second intervention to tDCS for the treatment of FM pain. Kang et al. [29] found that adding pharmacotherapy (Pregabalin or Duloxetine) to tDCS over M1 enhanced the analgesic effect compared to tDCS alone or pharmacotherapy alone. Silva et al. [35] reported that adding a Go/No-go task to tDCS over DLPFC improved attention and pain in FM patients, suggesting a possible role of cognitive modulation. Yoo et al. [6] showed that adding prefrontal tDCS before occipital nerve stimulation (ONS) increased pain relief and quality of life in FM patients who did not respond to ONS alone. Mendonca et al. [20] demonstrated that adding aerobic exercise (AE) to tDCS over M1 reduced pain intensity and improved mood and anxiety in FM patients, indicating a synergistic effect of both interventions. These studies suggest that combining tDCS with other interventions may optimize the analgesic responses in FM, but further research is needed to compare the efficacy and safety of these different strategies.

### New approaches

Two studies [22, 31] employed modifications to conventional tDCS interventions that demonstrated significant effects in reducing pain intensity when compared to sham groups. Villamer et al. [22] applied high-definition transcranial direct current stimulation (HD-tDCS) to offer a more precise and focused method of stimulation for a single session. Brietzke et al. [31] utilized home-based tDCS as a novel approach, which monitored treatment adherence by recording impedance, time of use, and current flow.

### Synthesis of results

#### Overall analysis

**Fig. 2:**
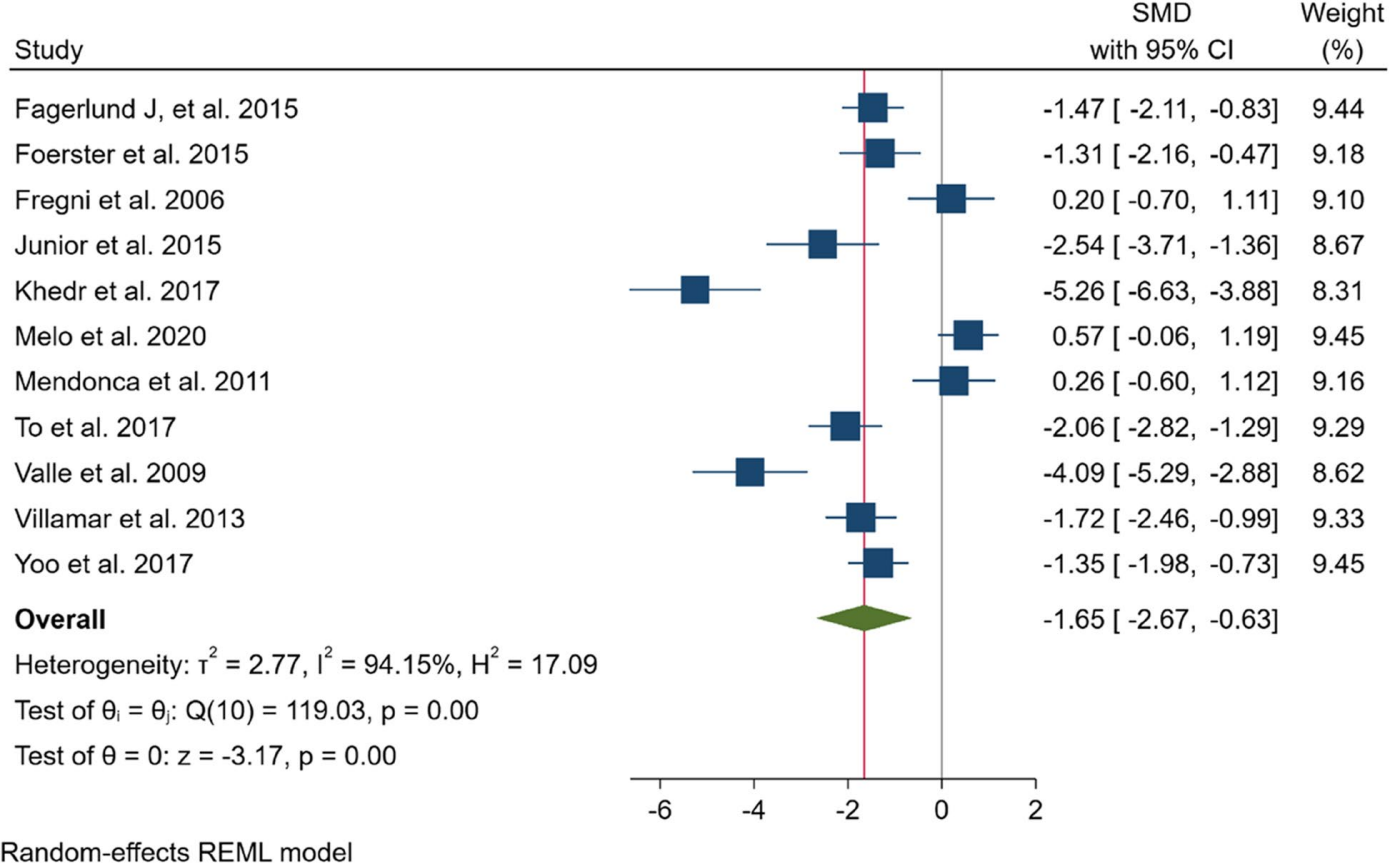
Meta-analysis of the effect of anodal tDCS on pain intensity in fibromyalgia for all included studies

**Fig. 3:**
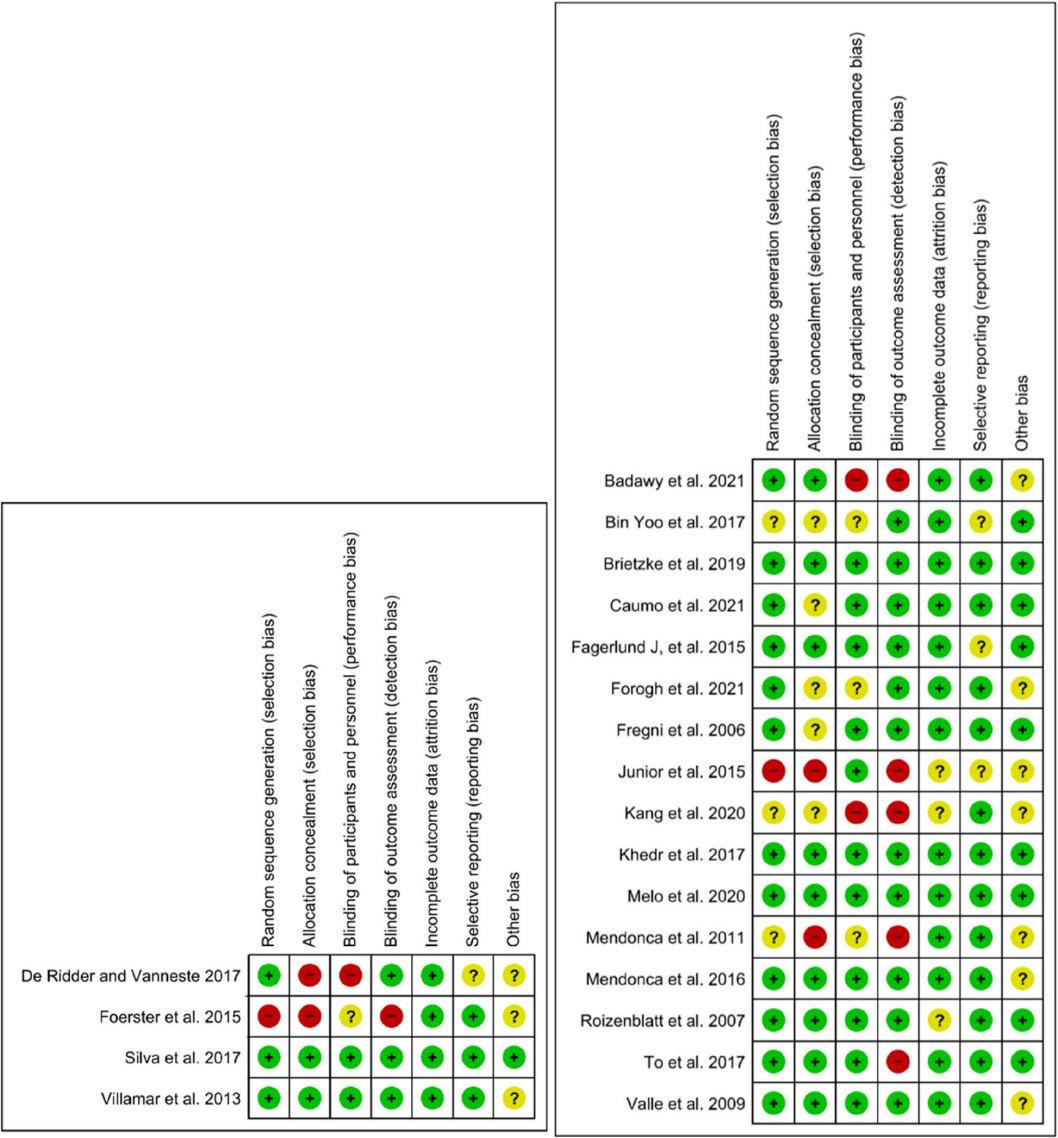
Risk of bias of included study. Randomized clinical trial (left) and crossover trial (right) based on authors’ judgment

**Fig. 4:**
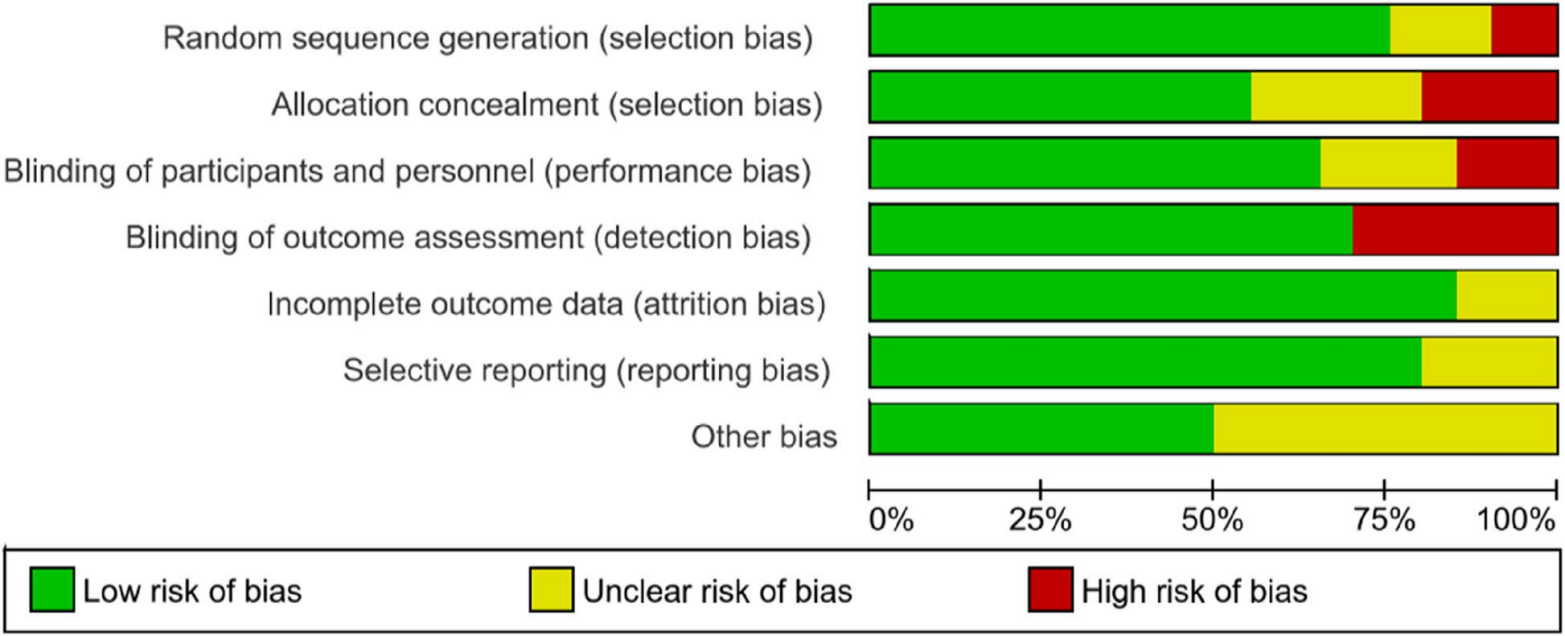
Risk of bias assessment based on subscales for all included studies based on authors’ judgment

**Fig. 5:**
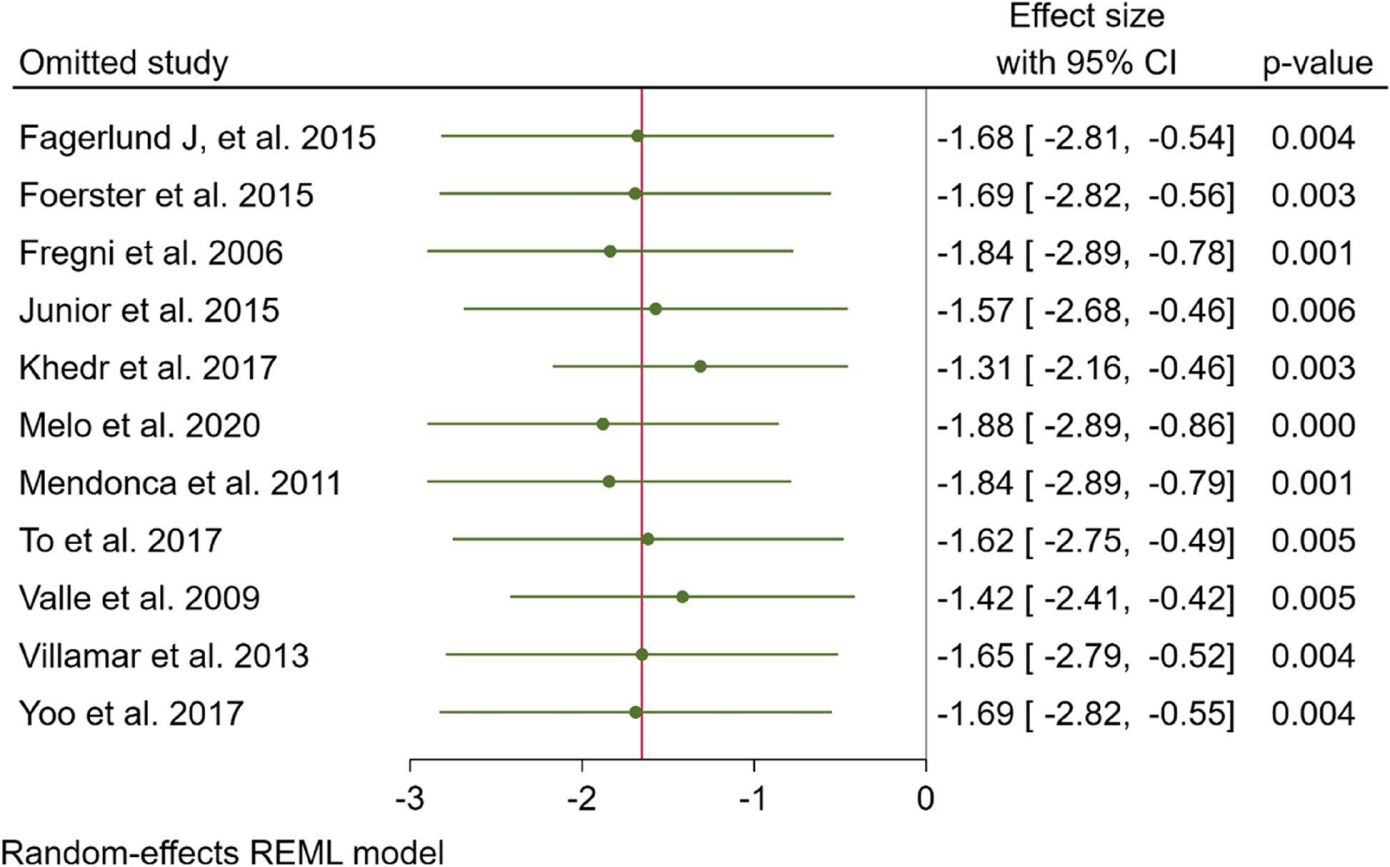
sensitivity plots of all included study

**Fig. 6:**
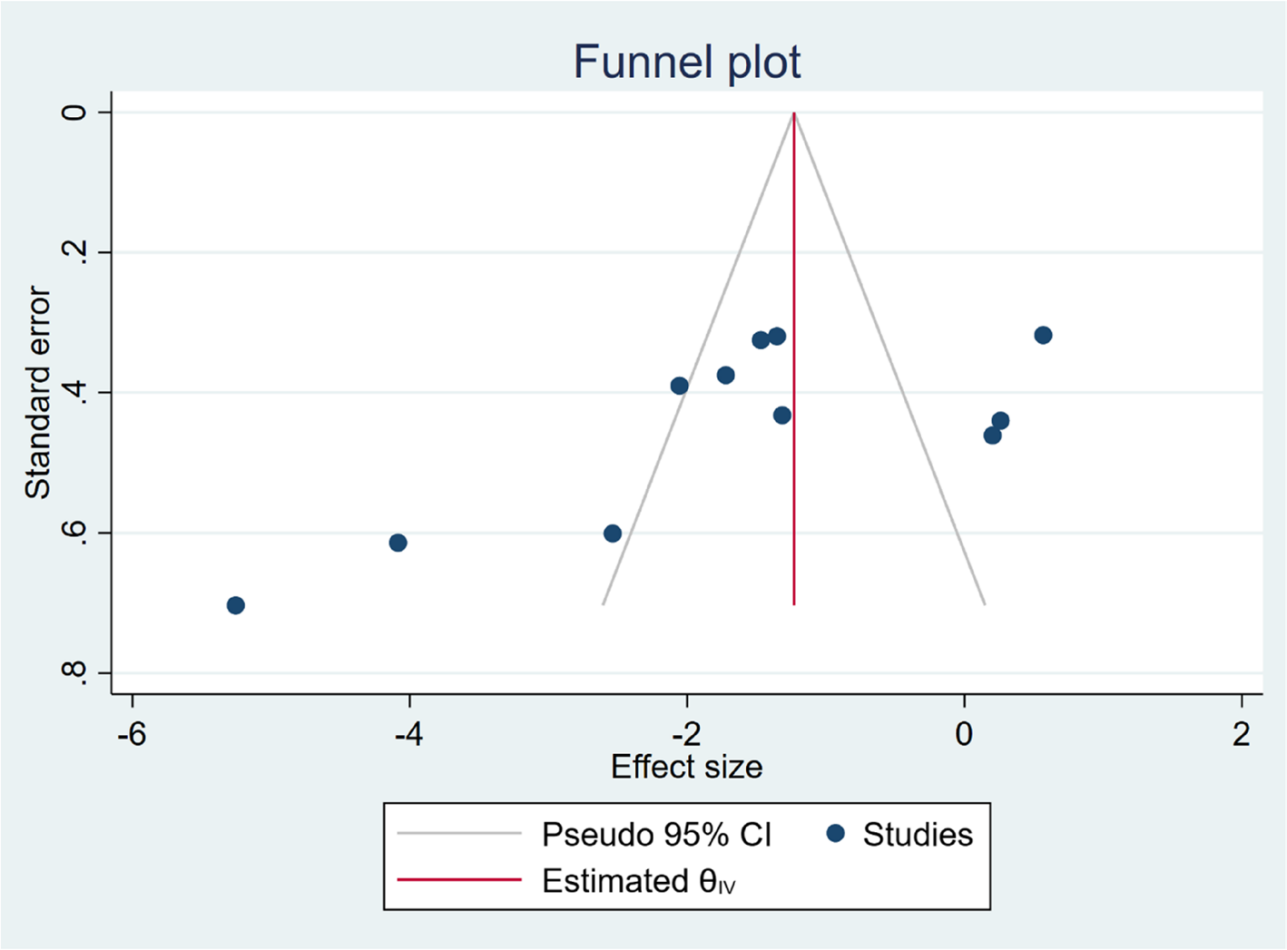
Funnel plot of all included study

We conducted a meta-analysis of 11 studies [6, 8, 18, 19, 21, 22, 26, 28, 30, 33, 34] with a total sample size of 414 subjects to compare the effects of tDCS and control interventions on pain intensity in patients with fibromyalgia. We aggregated 22 effect sizes and determined that tDCS significantly reduced pain intensity compared to controls (SMD = -1.65; 95% CI -2.67 to -0.63). However, we also identified significant heterogeneity (I2 = 94.16%) among the studies (see Fig. 2). Therefore, we conducted subgroup analysis to investigate potential sources of heterogeneity (please refer to Supplementary Material Part A, Figs. 1, 2, 3, 4, 5 and 6).

#### Risk of bias within studies

We assessed the quality and risk of bias of the included studies using the Cochrane risk-of-bias tool. Random sequence generation (selection bias) was low in 15 studies, two had a high risk, and three had an unclear risk. Allocation concealment (selection bias) was deemed low in 11, high in four, and unclear in five studies. Performance bias and detection bias were reported as high in three and six studies, respectively. On average, the quality assessments indicated that the studies had a low risk of bias (Figs. 3 and 4).

#### Sensitivity analysis and publication bias

We conducted a sensitivity analysis to evaluate the individual impact of each study on the SMD, which serves as the primary outcome in our mathematical model. This analysis involved the systematic removal of one study at a time. The findings, depicted in Fig. 5, indicated that the exclusion of Khedr et al.‘s study [30] had a comparatively greater influence on the estimation of the overall effect size when compared to the other studies (effect size = -1.31; 95% CI -2.16 to -0.46, *p* = 0.003). However, it is important to note that this particular study carried a relatively low weight in the meta-analysis, accounting for only 8.31% of the total, and as a result, its impact on both the SMD and the 95% CI was limited. Additionally, we conducted an examination of publication bias employing Egger’s test, Begg’s test, and a funnel plot. The funnel plot displayed an asymmetric distribution of the data, implying the potential presence of publication bias. Nonetheless, this observation contradicted the outcomes of Egger’s and Begg’s tests, which indicated a weak risk of publication bias (*p* = 0.001 and *p* = 0.06, respectively) as illustrated in Fig. 6 Consequently, we conducted the trim-and-fill method, which ultimately revealed no evidence of publication bias.

#### Subgroup analysis

Subgroup analyses were conducted based on variations in research design, the pain assessment tool employed, the number of tDCS sessions administered, current intensity, electrode placement, and gender. The analysis, as depicted in Table 2, revealed an improvement in pain scores across both types of included study designs, namely randomized controlled trials (RCT) (SMD = -1.70, 95% CI [-2.97, -0.42]) and crossover studies (SMD = -1.55, 95% CI [-2.10, -0.99]), with no statistically significant difference observed (*p* = 0.83). Upon further examination, the analysis stratified by the pain assessment measurement tools demonstrated a significant disparity in pain scores when utilizing the Visual Analog Scale (VAS) (SMD = -1.97, 95% CI [-3.53, -0.41]) and the Numeric Rating Scale (NRS) (SMD = -1.18, 95% CI [-2.11, -0.25]), yet no significant distinction between the two tools was evident. Likewise, the subgroups involving the primary motor cortex (M1) (SMD = -1.13, 95% CI [-2.75, 0.49]) and dorsolateral prefrontal cortex (DLPFC) (SMD = -4.19, 95% CI [-9.01, 0.63]) exhibited a significant effect in reducing pain, with no discernible variation between them. Regarding current intensity, both 2 mA (SMD = -1.55, 95% CI [-2.97, -0.13]) and less than 2 mA (SMD = -1.88, 95% CI [-2.67, -1.19]) were associated with lower pain scores. Furthermore, an analysis based on gender revealed a noteworthy reduction in pain following intervention in both male and female groups, as well as in the female-only subgroup (SMD = -1.71, 95% CI [-3.35, -0.08] and SMD = -1.29, 95% CI [-2.68, 0.10], respectively). In the encompassed studies, the number of sessions varied, including 1, 5, 8, and 10 sessions, all of which exhibited a significant reduction in pain. However, no statistically significant distinctions were identified among these session counts. Detailed information regarding the subgroup analyses can be found in Table 2, while the forest plots are available in Supplementary Material Part A, Figs. 1, 2, 3, 4, 5 and 6.

**Table 2:** Meta-analysis of the subgroups of the included studies

Subgroups	Number of studies	Standardized mean difference (95% CI)	*P*-value
Type of Study			
RCT	9	-1.70 (-2.97, -0.42)	< 0.001
Crossover study	2	-1.55 (-2.10, -0.99)	0.48
Test of group differences: Q_b_ (1) = 0.04, *p* = 0.83			
Pain assessment tool			
VAS	7	-1.97 (-3.53, -0.41)	< 0.001
NRS	4	-1.18 (-2.11, -0.25)	< 0.001
Test of group differences: Q_b_ (1) = 0.73, *p* = 0.39			
Number of tDCS sessions			
10 sessions	4	-2.87 (-5.29, -2.88)	< 0.001
8 sessions	2	-1.67 (-2.35, -0.98)	0.16
5 sessions	4	-0.43 (-1.59, 0.74)	< 0.001
1 session	2	-0.74 (-2.69, 1.20)	< 0.001
Test of group differences: Q_b_ (3) = 5.22, *p* = 0.16			
Current intensity			
2 mA	8	-1.55 (-2.97, -0.13)	< 0.001
Less than 2 mA	3	-1.88 (-2.67, -1.19)	0.18
Test of group differences: Q_b_ (1) = 0.17, *p* = 0.68			
Electrode Position			
M1	9	-1.13 (-2.75, 0.49)	< 0.001
DLPFC	3	-4.19 (-9.01, 0.63)	< 0.001
Test of group differences: Qb (1) = 1.39, *p* = 0.24			
Sexuality			
Only Female	6	-1.29 (-2.68, 0.10)	< 0.001
Female & Male	6	-1.71 (-3.35, -0.08)	< 0.001
Test of group differences: Q_b_ (1) = 0.15, *p* = 0.70			

*Abbreviations*: *CI *Confidence interval; *RCT *Randomized control trial, *VAS *Visual analogue scale, *NRS *Numeric rating scale, *M1 *Primary motor cortex, *DLPFC *Dorsolateral prefrontal cortex

## Discussion

The present systematic review included 20 studies, of which 11 were eligible for quantitative analysis. Nine of these were RCTs, and two were within-subject crossovers. The meta-analysis revealed that active tDCS reduced pain intensity in FM patients compared to the sham intervention. Both M1 and DLPFC, as the most frequently targeted regions in neuromodulation for pain processing, exhibited a significant decrease in pain intensity when stimulated. M1 emerged as the most commonly targeted site in the studies; nevertheless, stimulating both M1 and DLPFC proved effective in modulating pain intensity in FM. Our subgroup analysis failed to detect any differences between M1 and DLPFC.

The effect of more frequent sessions of tDCS on primary outcomes was not observed in the subgroup analysis. Although more frequent sessions of tDCS had the largest effect size in reducing pain (SMD = -2.69; 95% CI -5.37 to -0.02, *p* < 0.001), the test of group difference with less frequent sessions was not significant (*p* = 0.22). This finding contradicts the previously mentioned cumulative effect of tDCS on pain intensity reduction [31]. This controversy may exist because we primarily included studies that focused on short-term effects, and the longer-lasting effects of tDCS on pain intensity in the patient population need to be addressed in future studies.

Comparing the use of different pain intensity measures showed that NRS and VAS scales had no significant differences in depicting pain reduction in the intervention group compared to sham (*p*-value < 0.001 with a standardized mean difference of -2.02 for VAS and − 1.18 for NRS). Subgroup analysis revealed that the current intensity of 2 mA, as employed in the majority of included studies (8 out of 11), effectively reduced pain in the intervention group compared to the sham. However, there was no discernible group difference between 2 mA and protocols with current intensities less than 2 mA. Gender dependency regarding the analgesic effects of tDCS was explored in a subgroup analysis, where female patients exhibited a significant response to treatment. Nevertheless, no significant difference was observed when male patients were included in the studies (*p* = 0.75).

Most of the included studies reported either mild side effects or no side effects at all. The side effects that were most frequently reported include skin redness, sleepiness, transient sleep disturbances, itching, tingling, light headaches, and dizziness, demonstrating the safety and tolerability of this procedure. However, despite the consensus on the safety of tDCS, some studies (5 out of 20) reported significant side effects. This necessitates further research to quantitatively assess the side effects and offer guidance on the cost-effectiveness of decisions in clinical settings.

Several systematic reviews and meta-analyses have attempted to investigate the analgesic effect of neuromodulation on chronic pain. Xiong et al. reviewed the current state of the art and future directions of non-invasive brain stimulation (NIBS) for assisting individuals with chronic pain. They illustrated a growing trend in the research field of NIBS over the last 20 years, demonstrating that Repetitive Transcranial Magnetic Stimulation (rTMS) and tDCS are surpassing other neuromodulation methods, with tDCS even surpassing rTMS. They deliberated upon the mechanisms, applications, and challenges associated with various NIBS techniques and summarized the evidence from clinical trials and meta-analyses regarding the efficacy and safety of NIBS for various chronic pain conditions, such as neuropathic pain, fibromyalgia (FM), migraine, and low back pain [36]. Clinical and experimental studies suggest that rTMS may reduce pain in FM patients by modulating neural pain pathways, such as the descending inhibitory pathways and brain regions involved in social-affective functions, such as the right temporal lobe [36]. El-Badawy et al. and Forogh et al. compared the effects of rTMS and tDCS on pain intensity in patients with FM. They reported that both rTMS and tDCS significantly reduced pain intensity in FM patients, with the rTMS group experiencing greater and longer-lasting effects [25, 27].

Wen et al. conducted a systematic review and meta-analysis to evaluate the effects of tDCS on pain, depression, and anxiety symptoms in patients with chronic pain. They included 27 randomized controlled trials with a total of 1,015 participants who received tDCS or sham stimulation for various chronic pain conditions. They found that tDCS was significantly more effective than sham stimulation in reducing short-term pain intensity (SMD = -0.43, 95% CI = -0.75 to -0.12), short-term and middle-term depression (SMD = -0.31, 95% CI = -0.47 to -0.14, and SMD = -0.35, 95% CI = -0.58 to -0.11), and anxiety scores (SMD = -0.36, 95% CI = -0.58 to -0.14) in patients with chronic pain, but longer-lasting effects were not observed [37].

Our findings are consistent with previous reviews and meta-analyses, which have also reported a significant analgesic effect of tDCS in FM [9, 12–14, 38–41]. Hou et al. [12] incorporated 16 studies and endeavored to investigate the effects of rTMS and tDCS as supplementary treatments for FM. The study uncovered that NBS yielded significantly advantageous outcomes in terms of pain reduction, alleviation of depression, mitigation of fatigue, amelioration of sleep disturbance, and enhancement of general health/functionality in FM patients. Additionally, the study revealed that rTMS exhibited a more pronounced effect size when compared to tDCS. Furthermore, within the realm of pain reduction, M1 stimulation demonstrated a subtle but greater effect size than DLPFC stimulation, whereas DLPFC stimulation exhibited a subtle but greater effect size in terms of depression improvement when compared to M1 stimulation. In a similar vein, Zhu et al. conducted a meta-analysis encompassing a review of 6 RCTs and identified the efficacy of tDCS, albeit exclusively when the target region was M1, as opposed to DLPFC [13]. Another study found a significant effect size in pain reduction when comparing stimulation of the M1 area to the DLPFC [41]. Lloyd et al. found that tDCS was significantly superior to sham in reducing pain (*p*-value = 0.005 with an SMD of -0.5; 95% confidence interval − 0.4 to 0.62). They concluded that active anodal tDCS, with a current intensity of 2 mA applied to the left M1 for 20 min per session over 10 sessions, was the most effective approach for alleviating pain in FM [38]. The two most recent meta-analyses on the matter by Cheng et al. and Teixeira et al. reported a standardized mean difference of 0.4990 (95% CI = 0.1757–0.8223, *p* < 0.01) and 1.22 (95% CI = 0.80–1.65, *p* < 0.001), respectively, in pain reduction among FM patients through the administration of tDCS.

However, our review also differs from previous ones in some aspects of the methods and results. First, we included more studies in our meta-analysis because we searched additional databases and updated the search until June 2022. This augmentation enhanced both the quantity and quality of studies, thereby diminishing the risk of publication bias. Second, we conducted a subgroup analysis based on the target site of tDCS, owing to the identification of substantial heterogeneity among the studies. We ascertained that both M1 and DLPFC stimulation were efficacious in mitigating pain in FM patients, whereas some of the prior reviews failed to detect a significant effect of DLPFC stimulation [9, 13]. Third, we conducted a meta-analysis to investigate the potential factors that influence the effect size of tDCS on pain outcomes, such as current intensity, target location, number of sessions, study design, the subject’s gender, and the pain measurement scale. We did not identify any significant associations between these factors and the outcomes.

One explanation is that tDCS has neurochemical effects and alters the levels of neurotransmitters such as glutamate, glutamine, GABA, N-Acetyl Aspartate (NAA), and endorphins, all of which are implicated in pain transmission and modulation. Through the augmentation of anodal stimulation in M1 or other cerebral regions, tDCS may potentially amplify the secretion of inhibitory neurotransmitters and endogenous opioids while diminishing the release of excitatory neurotransmitters, thereby yielding decreased pain sensitivity and increased pain tolerance in FM patients [26, 30].

Another explanation is that tDCS modulates the functional connectivity and activity of brain regions and networks that are involved in pain processing and modulation. tDCS stimulation lacks focality, and studies have shown that the stimulation usually spreads beyond the target site, thus resulting in network-wide changes [42]. Cummiford et al. found that repetitive tDCS stimulation of M1 will alter the resting state functional connectivity in FM patients. The insula, anterior cingulate cortex, thalamus, and somatosensory cortex are among the brain regions where changes in functional connectivity are reported. These changes might reflect neuroplasticity induced by tDCS and could be explained by lasting pain relief beyond the stimulation period [43].

A third explanation is that tDCS interacts with the individual’s brain state, such as their mood, attention, motivation, cognitive load, and expectations, all of which can influence the efficacy and outcome of tDCS on pain modulation [44, 45]. By combining tDCS with other interventions such as aerobic exercise, cognitive-behavioral therapy, or task-oriented approaches, tDCS may improve brain-state dependency and optimize the analgesic effect of tDCS in FM patients [46]. These explanations are not mutually exclusive and may work together to produce a cumulative analgesic effect of tDCS in FM patients. However, more research is needed to confirm the exact mechanisms and optimal parameters of tDCS for pain management in FM.

Our systematic review harbors some limitations that necessitate acknowledgment and remediation. Firstly, there exists a risk of bias within the included studies, given that a majority of them exhibited ambiguity or a high risk of bias in specific domains, notably randomization, allocation concealment, blinding, and incomplete outcome data. Such issues have the potential to compromise the internal validity and reliability of the studies, thereby affecting the accuracy and precision of the results derived from the meta-analysis. Secondly, the sample sizes across the included studies were notably diminutive, ranging from 10 to 60 participants per study. This diminishment could impede statistical power and the generalizability of the findings, consequently augmenting heterogeneity and fostering uncertainty in the results of the meta-analysis. Thirdly, the paucity of long-term follow-ups within the majority of studies precluded our ability to assess the durability and persistence of tDCS effects on pain outcomes. This insufficiency could curtail the clinical relevance and practicality of employing tDCS for managing pain in patients with fibromyalgia who require prolonged treatment. Lastly, the variability in outcome measures and stimulation protocols among studies posed a substantial challenge in the comparison and synthesis of tDCS effects on pain outcomes. Disparate outcome measures might capture distinct facets of pain and quality of life among fibromyalgia patients, each possessing unique psychometric attributes and responsiveness to change. Meanwhile, dissimilar stimulation protocols could potentially exert divergent mechanisms of action, impacting cortical excitability, neurotransmission, neural networks, and brain-state dependency within fibromyalgia patients. Furthermore, these protocols might also introduce varying safety and feasibility considerations. Regrettably, these aspects were not comprehensively investigated and reported in the existing literature, rendering any conclusive determination unattainable.

In consideration of the limitations of our study, we acknowledge that the shortcomings of high-quality research on the topic, heterogeneous study designs, the lack of generalizability of mechanistic surveys, and the absence of investigations into long-term effects in previous studies may have implications for the generalizability of our findings.

Our systematic review bears significant implications for clinical practice and forthcoming research on tDCS for FM. Firstly, tDCS appears to constitute a viable and secure treatment option for FM patients, as the majority of studies reported either no or mild adverse effects alongside high adherence rates. Nonetheless, additional investigations are imperative to assess the long-term safety and tolerability of tDCS, especially in the context of home-based or self-administered protocols, which may extend the cumulative exposure time. Secondly, there exists an exigency for more standardized and individualized treatment protocols for tDCS, given the marked variability in stimulation parameters and target sites observed across studies. Future inquiries should employ rigorous methodologies to ascertain the optimal current intensity, duration, frequency, and electrode montage tailored to each patient, contingent upon their pain characteristics and brain state. Thirdly, tDCS may potentially yield synergistic effects when concomitantly administered with other interventions or modalities, such as pharmacotherapy, cognitive-behavioral therapy, exercise, or neurofeedback. Furthermore, adopting a holistic approach to pain management, which takes into account the affective and cognitive facets of pain when designing a tDCS regimen and selecting target site(s), is poised to augment its efficacy. As underscored in this review study, we advocate for an augmentation in sample sizes to bolster the robustness of investigations, the exploration of longer-lasting effects of the proposed interventions to address the issue of chronic pain, and a meticulous documentation and rigorous characterization of side effects as potential focal points for future studies contributing to the field.

## Conclusion

tDCS is a promising and clinically sound treatment for chronic pain conditions, such as fibromyalgia, believed to originate from the central nervous system (CNS). However, we did not identify a superior stimulation protocol in our subgroup analysis. More experimental studies are required to investigate the fundamentals of the brain changes induced by various neurostimulation modalities and the brain mechanisms underlying their effects, possibly by incorporating neuro-electrophysiological or neuroimaging studies in conjunction with the intervention. Future research should explore the potential benefits of combining tDCS with other interventions or modalities for fibromyalgia patients. Lastly, it is imperative to identify the most effective target sites and optimal stimulation parameters within individualized treatment protocols that take into account the brain-state dependency of neurostimulation modalities before embarking on further large-scale randomized controlled trials.

### Supplementary Information

**Additional file 1: Part A: Subgroup analyses. Figure S1.** Subgroup meta-analysis of the included studies for assessing the effect of  transcranial Direct-Current Stimulation (tDCS) on pain intensity of Fibromyalgia patients based on the type of study. **Figure S2.** Subgroup meta-analysis of the included studies for assessing the effect of  transcranial Direct-Current Stimulation (tDCS) on pain intensity of Fibromyalgia patients based on the pain assessment tool. **Figure S3.** Subgroup meta-analysis of the included studies for assessing the effect of  transcranial Direct-Current Stimulation (tDCS) on pain intensity of Fibromyalgia patients based on number of tDCS sessions. **Figure S4.** Subgroup meta-analysis of the included studies for assessing the effect of  transcranial Direct-Current Stimulation (tDCS) on pain intensity of Fibromyalgia patients based on current intensity (mA). **Figure S5.** Subgroup meta-analysis of the included studies for assessing the effect of  transcranial Direct-Current Stimulation (tDCS) on pain intensity of Fibromyalgia patients based on electrode position (anode). **Figure S6.** Subgroup meta-analysis of the included studies for assessing the effect of  transcranial Direct-Current Stimulation (tDCS) on pain intensity of Fibromyalgia patients based on sexuality. **Part B: Search Strategy**

## Data Availability

All information required is given in the text and supplementary materials, other supplementary information can be obtained upon email from the corresponding author.
