# Supplementary material for: The effects of transcranial direct-current stimulation (tDCS) on pain intensity of patients with fibromyalgia: a systematic review and meta-analysis
Source: BMC Neurol. 2023 Nov 2;23:395. doi: 10.1186/s12883-023-03445-7 (PMC10621179; doi:10.1186/s12883-023-03445-7)
Supplement: Supplementary file 1 — Additional file 1: Part A: Subgroup analyses. Figure S1. Subgroup meta-analysis of the included studies for assessing the effect of transcranial Direct-Current Stimulation (tDCS) on pain intensity of Fibromyalgia patients based on the type of study. Figure S2. Subgroup meta-analysis of the included studies for assessing the effect of transcranial Direct-Current Stimulation (tDCS) on pain intensity of Fibromyalgia patients based on the pain assessment tool. Figure S3. Subgroup meta-analysis of the included studies for assessing the effect of transcranial Direct-Current Stimulation (tDCS) on pain intensity of Fibromyalgia patients based on number of tDCS sessions. Figure S4. Subgroup meta-analysis of the included studies for assessing the effect of transcranial Direct-Current Stimulation (tDCS) on pain intensity of Fibromyalgia patients based on current intensity (mA). Figure S5. Subgroup meta-analysis of the included studies for assessing the effect of transcranial Direct-Current Stimulation (tDCS) on pain intensity of Fibromyalgia patients based on electrode position (anode). Figure S6. Subgroup meta-analysis of the included studies for assessing the effect of transcranial Direct-Current Stimulation (tDCS) on pain intensity of Fibromyalgia patients based on sexuality. Part B: Search Strategy [file 12883_2023_3445_MOESM1_ESM.docx]

**Supplementary materials**

Table of Contents

[Part A: Subgroup analyses 2](#_Toc137506811)

[Figure S1. Subgroup meta-analysis of the included studies for assessing the effect of transcranial Direct-Current Stimulation (tDCS) on pain intensity of Fibromyalgia patients based on the type of study 2](#_Toc137506812)

[Figure S2. Subgroup meta-analysis of the included studies for assessing the effect of transcranial Direct-Current Stimulation (tDCS) on pain intensity of Fibromyalgia patients based on the pain assessment tool 3](#_Toc137506813)

[Figure S3. Subgroup meta-analysis of the included studies for assessing the effect of transcranial Direct-Current Stimulation (tDCS) on pain intensity of Fibromyalgia patients based on the number of tDCS sessions 4](#_Toc137506814)

[Figure S4. Subgroup meta-analysis of the included studies for assessing the effect of transcranial Direct-Current Stimulation (tDCS) on pain intensity of Fibromyalgia patients based on current intensity (mA) 5](#_Toc137506815)

[Figure S5. Subgroup meta-analysis of the included studies for assessing the effect of transcranial Direct-Current Stimulation (tDCS) on pain intensity of Fibromyalgia patients based on electrode position (anode) 6](#_Toc137506816)

[Figure S6. Subgroup meta-analysis of the included studies for assessing the effect of transcranial Direct-Current Stimulation (tDCS) on pain intensity of Fibromyalgia patients based on sexuality 7](#_Toc137506817)

[Part B: Search Strategy 8](#_Toc137506818)

# **Part A: Subgroup analyses**


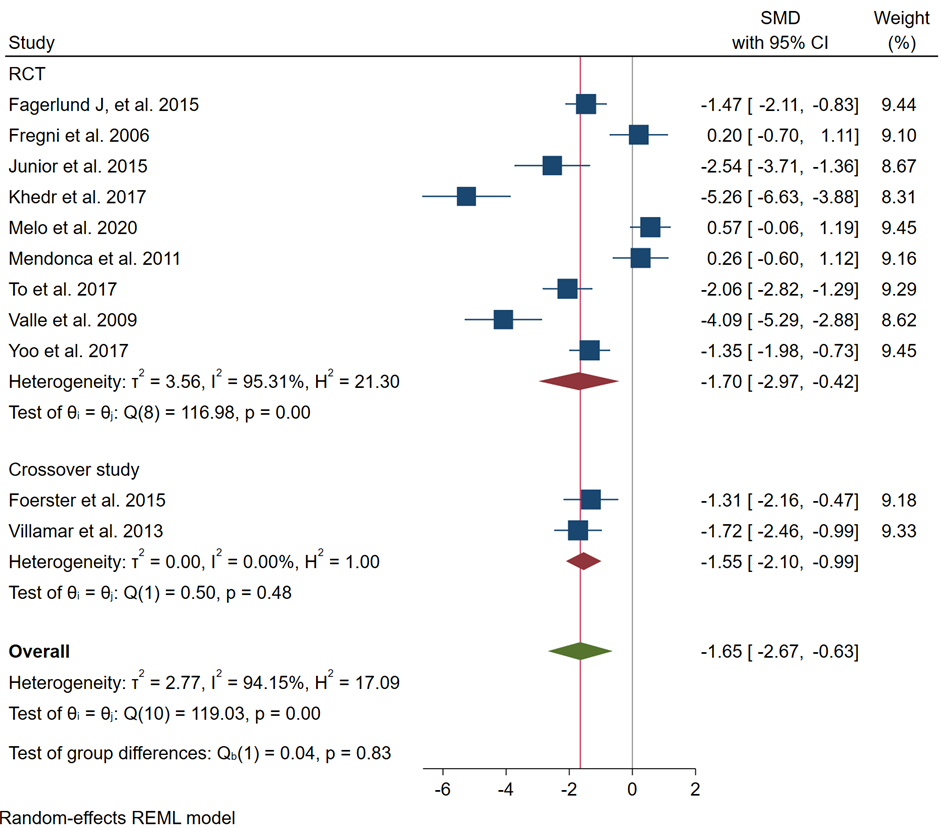


## Figure S1. Subgroup meta-analysis of the included studies for assessing the effect of transcranial Direct-Current Stimulation (tDCS) on pain intensity of Fibromyalgia patients based on the type of study


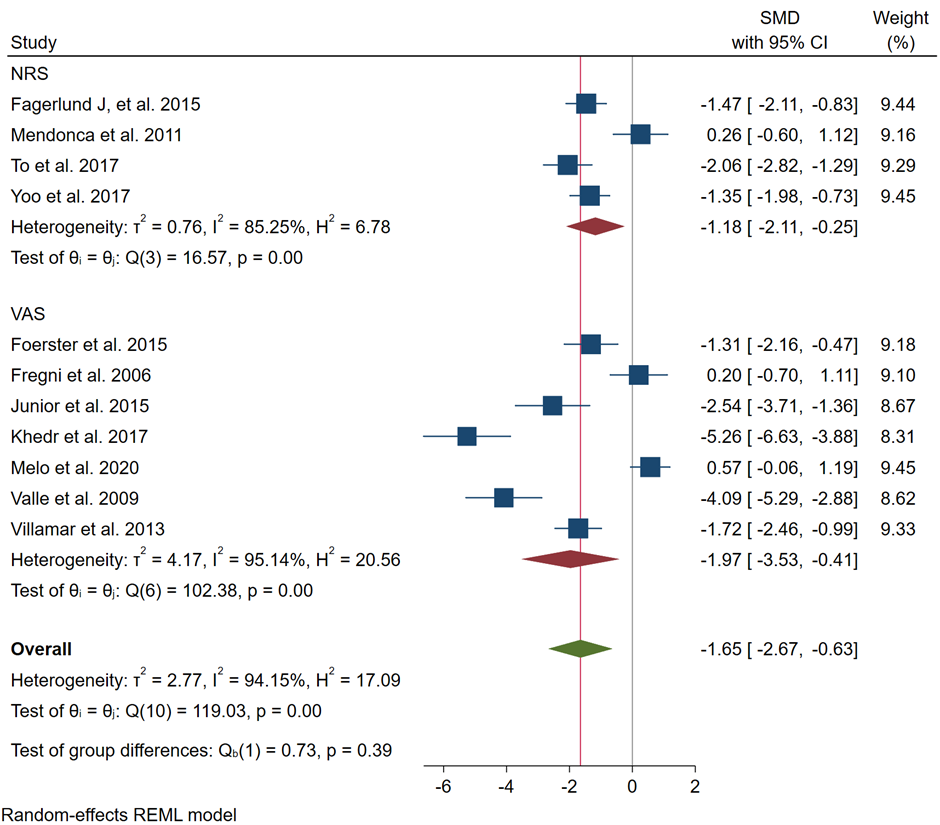


## Figure S2. Subgroup meta-analysis of the included studies for assessing the effect of transcranial Direct-Current Stimulation (tDCS) on pain intensity of Fibromyalgia patients based on the pain assessment tool


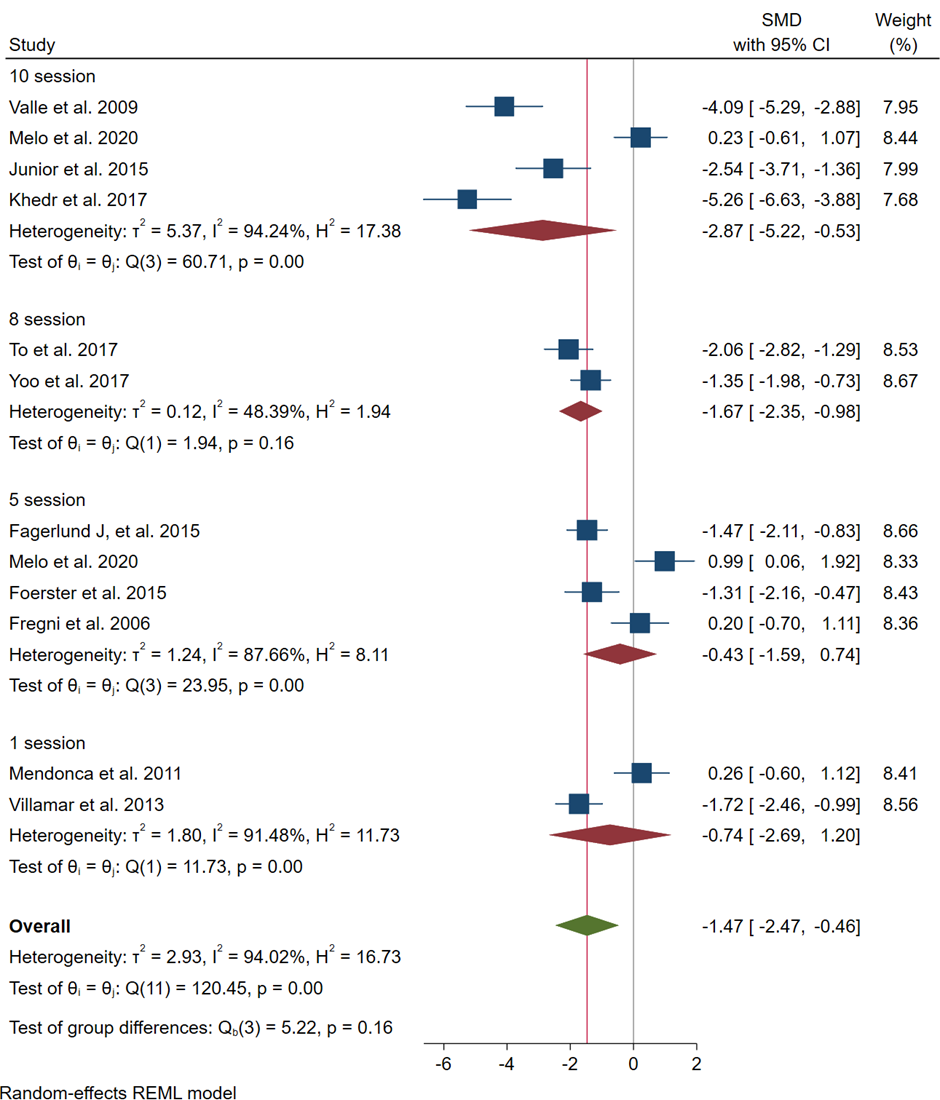


## Figure S3. Subgroup meta-analysis of the included studies for assessing the effect of transcranial Direct-Current Stimulation (tDCS) on pain intensity of Fibromyalgia patients based on number of tDCS sessions


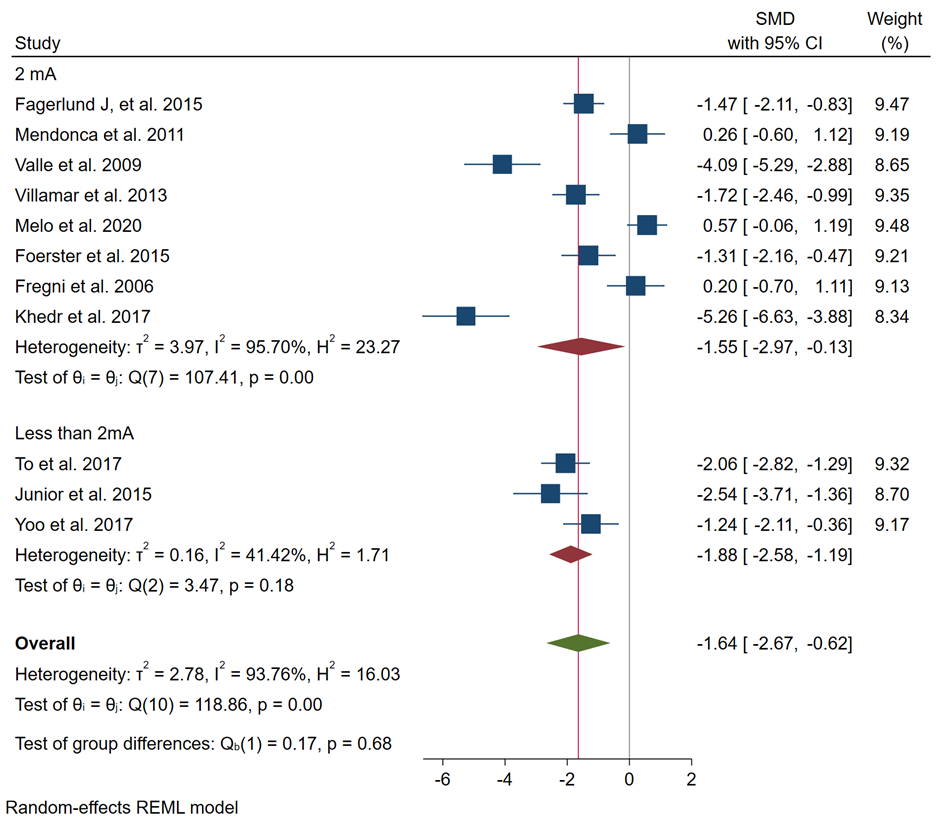


## Figure S4. Subgroup meta-analysis of the included studies for assessing the effect of transcranial Direct-Current Stimulation (tDCS) on pain intensity of Fibromyalgia patients based on current intensity (mA)


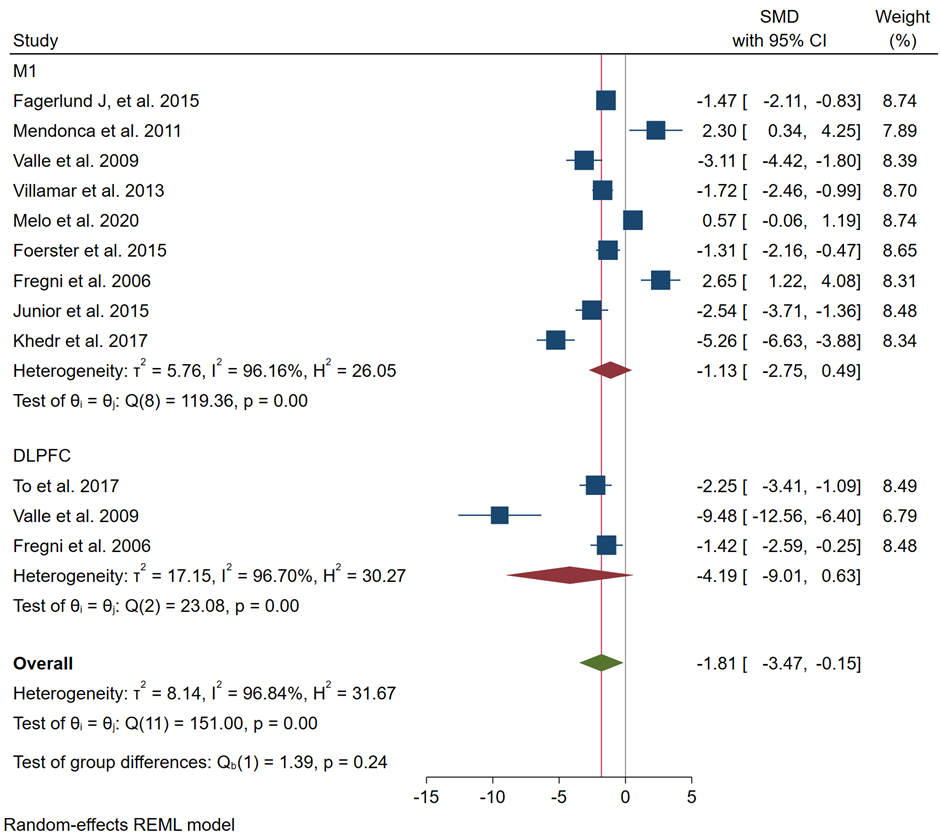


## Figure S5. Subgroup meta-analysis of the included studies for assessing the effect of transcranial Direct-Current Stimulation (tDCS) on pain intensity of Fibromyalgia patients based on electrode position (anode)


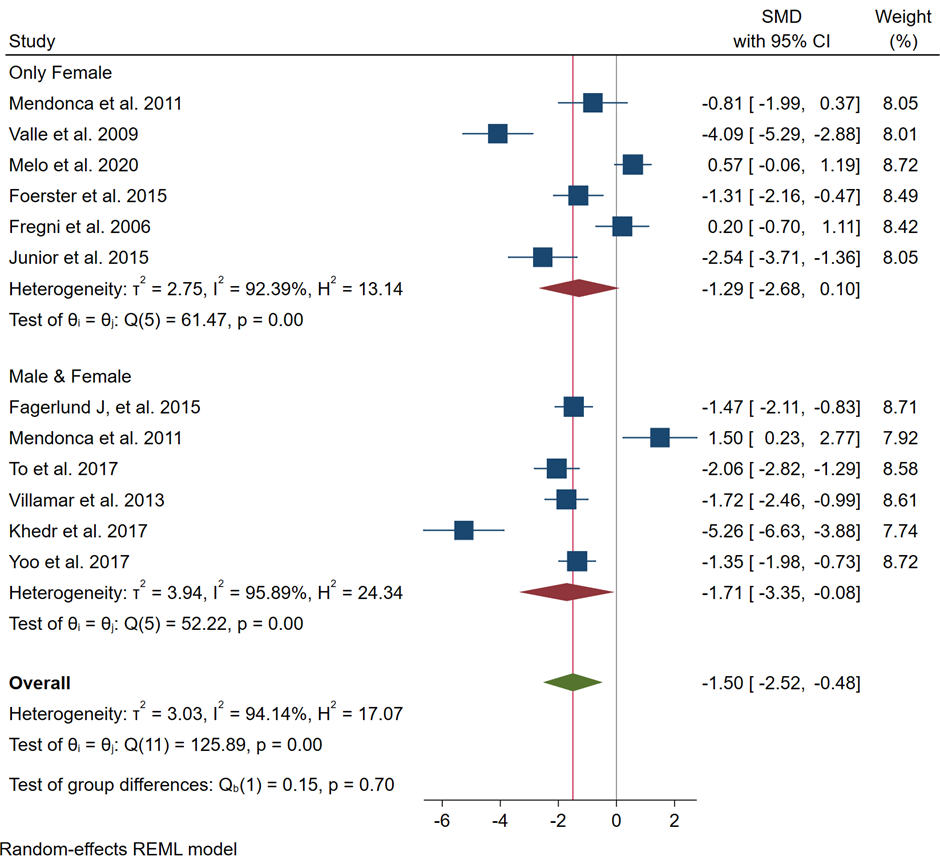


## Figure S6. Subgroup meta-analysis of the included studies for assessing the effect of transcranial Direct-Current Stimulation (tDCS) on pain intensity of Fibromyalgia patients based on sexuality

# **Part B: Search Strategy**

**Databases:**

1. **PubMed**: 73 results

1: ((((("Transcranial Direct Current Stimulation"[Mesh]) OR (Transcranial Direct Current Stimulation[Title/Abstract])) OR (tDCS[Title/Abstract])) OR (Transcranial Electrical Stimulation[Title/Abstract])) OR (Cathodal Stimulation[Title/Abstract])) OR (anodal Stimulation[Title/Abstract])

#2: ((((("Fibromyalgia"[Mesh]) OR (Fibromyalg*[Title/Abstract])) OR (Muscular Rheumatism[Title/Abstract])) OR (Fibrosit*[Title/Abstract])) OR (Diffuse Myofascial Pain Syndrome[Title/Abstract])) OR (Fibromyosit*[Title/Abstract])

#3: (((((("Fibromyalgia"[Mesh]) OR (Fibromyalg*[Title/Abstract])) OR (Muscular Rheumatism[Title/Abstract])) OR (Fibrosit*[Title/Abstract])) OR (Diffuse Myofascial Pain Syndrome[Title/Abstract])) OR (Fibromyosit*[Title/Abstract])) AND (((((("Transcranial Direct Current Stimulation"[Mesh]) OR (Transcranial Direct Current Stimulation[Title/Abstract])) OR (tDCS[Title/Abstract])) OR (Transcranial Electrical Stimulation[Title/Abstract])) OR (Cathodal Stimulation[Title/Abstract])) OR (anodal Stimulation[Title/Abstract]))

Final: #1 AND #2 AND #3

1. **Scopus**: 132 results

(TITLE-ABS-KEY(Fibromyalg*) OR TITLE-ABS-KEY(Muscular Rheumatism Fibromyositis) OR TITLE-ABS-KEY(Fibrosit*) OR TITLE-ABS-KEY(Diffuse Myofascial Pain Syndrome) OR TITLE-ABS-KEY(Fibromyosit*)) AND (TITLE-ABS-KEY(transcranial direct current) OR TITLE-ABS-KEY(tDCS) OR TITLE-ABS-KEY(Transcranial Electrical Stimulation) OR TITLE-ABS-KEY(Cathodal Stimulation) OR TITLE-ABS-KEY(anodal Stimulation))

1. **Web of sciences**: 94 results

#1: TS=( Fibromyalg* OR Muscular Rheumatism OR Fibrosit* OR Diffuse Myofascial Pain Syndrome OR Fibromyosit*)

#2: TS=( transcranial direct current stimulation OR tDCS OR Transcranial Electrical Stimulation OR Cathodal Stimulation OR anodal Stimulation)

Final: #1 AND #2

1. **Embase**: 97 results

#1: Fibromyalg*:ab,ti OR 'Muscular Rheumatism':ab,ti OR Fibrosit*:ab,ti OR 'Diffuse Myofascial Pain Syndrome':ab,ti OR Fibromyosit*:ab,ti

#2: 'transcranial direct current stimulation':ab,ti OR tDCS:ab,ti OR 'Transcranial Electrical Stimulation':ab,ti OR 'Cathodal Stimulation':ab,ti OR 'anodal Stimulation':ab,ti

Final: #1 AND #2

1. **PsycInfo**: 25 results

#1: TI Fibromyalg* OR TI Muscular Rheumatism OR TI Fibrosit* OR TI Diffuse Myofascial Pain Syndrome OR TI Fibromyosit* OR AB Fibromyalg* OR AB Muscular Rheumatism OR AB Fibrosit* OR AB Diffuse Myofascial Pain Syndrome OR AB Fibromyosit*

#2: TI transcranial direct current stimulation OR TI tDCS OR TI Transcranial Electrical Stimulation OR TI Cathodal Stimulation OR TI anodal Stimulation OR AB transcranial direct current stimulation OR AB tDCS OR AB Transcranial Electrical Stimulation OR AB Cathodal Stimulation OR AB anodal Stimulation

Final: #1 AND #2

1. **CINAHL** **complete**: 24 results

#1: TI Fibromyalg* OR TI Muscular Rheumatism OR TI Fibrosit* OR TI Diffuse Myofascial Pain Syndrome OR TI Fibromyosit* OR AB Fibromyalg* OR AB Muscular Rheumatism OR AB Fibrosit* OR AB Diffuse Myofascial Pain Syndrome OR AB Fibromyosit*

#2: TI transcranial direct current stimulation OR TI tDCS OR TI Transcranial Electrical Stimulation OR TI Cathodal Stimulation OR TI anodal Stimulation OR AB transcranial direct current stimulation OR AB tDCS OR AB Transcranial Electrical Stimulation OR AB Cathodal Stimulation OR AB anodal Stimulation

Final: #1 AND #2

1. **Cochrane**: 26 results

#1: Fibromyalg* in Title Abstract Keyword OR Fibrosit* in Title Abstract Keyword OR Muscular Rheumatism in Title Abstract Keyword AND Diffuse Myofascial Pain Syndrome in Title Abstract Keyword AND Fibromyosit* in Title Abstract Keyword

#2: transcranial direct current stimulation in Title Abstract Keyword OR tDCS in Title Abstract Keyword OR Transcranial Electrical Stimulation in Title Abstract Keyword OR Cathodal Stimulation in Title Abstract Keyword OR anodal Stimulation

Final: #1 AND #2
